# Supplementary material for: Monolithic electrostatic actuators with independent stiffness modulation
Source: Nat Commun. 2025 Jan 30;16:1174. doi: 10.1038/s41467-025-56455-z (PMC11782490; doi:10.1038/s41467-025-56455-z)
Supplement: Supplementary file 1 — Supplementary Information [file 41467_2025_56455_MOESM1_ESM.pdf]

Supplementary Information for

Monolithic Electrostatic Actuators with Independent Stiffness Modulation

Yuejun Xu[1], Jian Wen[1,2], Etienne Burdet[1], Majid Taghavi[1,3]*

[1] Department of Bioengineering, Imperial College London, London., UK.
[2] State Key Laboratory of Electrical Insulation and Power Equipment, Xi'an Jiaotong University, Xi'an, China.
[3] School of Engineering and Materials Science, Queen Mary University of London, London, UK

*Correspondence to: m.taghavi@imperial.ac.uk

Contents:

Supplementary Methods
Supplementary Figures
Supplementary Fig. 1: Comparison of deflection and bending stiffness of ERF beam
Supplementary Fig. 2: Isotonic test and comparison of static extension of an actuator
Supplementary Fig. 3: Effective stiffness and damping ratio form free response
Supplementary Fig. 4: Time response to a chirp signal excitation
Supplementary Fig. 5: Fabrication procedure of variable stiffness beams and actuators
Supplementary Fig. 6: Durability test
Supplementary Fig. 7: Example of a sinusoidal frequency chirp signal
Supplementary Fig. 8: Multilayer parallel capacitor for electrostatic contraction
Supplementary Fig. 9: Model of the ERF beam
Supplementary Fig. 10: Model of the actuator
Supplementary Fig. 11: The isometric test setup to measure force at a certain extension
Supplementary Fig. 12: Comparison of modelling and experimental results
Supplementary Fig. 13: Rheological behavior of ERF
Supplementary Fig. 14: Equivalent dynamic model of the ESRA
Supplementary Fig. 15: One-dimensional simplification of the agonist–antagonist system
Supplementary Fig. 16: Mechanical configuration of two ESRA in series
Supplementary Fig. 17: Comparison of three-point bending test result
Supplementary Tables
Supplementary Table 1: Comparison of key parameters between this works and some soft actuators
Supplementary Table 2: Comparison of key parameters between series ESRA and other stiffness and damping variation systems
Supplementary Table 3: Material properties of dielectric materials
Supplementary References

Supplementary Methods

Materials and components

The prototype of the ESRA consists of two identical beams. Supplementary Fig. 5a shows the fabrication process and materials of a beam: The electrodes are 12.7 mm wide steel strips (1.1274 carbon steel, H+S PRÄZISIONSFOLIEN GmbH, Geiman), and the insulator is PVC tape (AT7 PVC Electrical Insulation Tape, Advance Tapes, UK) with a width of 19 mm. The wires were soldered to the electrodes for voltage application. Encapsulation material should have a strong adhesive to prevent oil leakage; in this case, double-sided acrylic foam tape (3M™ VHB™ Adhesive Transfer Tapes, 3M, USA) is chosen for encapsulation. The diagram shows the insulation of the bottom electrode on both sides. In this case, only one electrode is fully encapsulated with wider PVC tape to ensure that no short circuit occurs. ERF (Ningbo Maiwei Technology, Co., LTD, China) is injected and encapsulated. The acrylic tape is cut into a rectangular frame by a cutter (Cricut Maker™ 3, Cricut, Inc.) and attached to an insulated electrode. The thickness of the ERF layer is changed by adjusting the thickness of the acrylic tape. ERF is injected into the rectangular channel, the volume of ERF should be as same as the volume of the channel. Then, the ERF is covered with another electrode and finally sealed with a 50-um acrylic tape to complete the encapsulation. Once the fabrication of the beam is completed, the actuator is assembled from two identical beams in a symmetric structure. Customized acrylic clips are held in place by plastic nuts and bolts, forming two hinge connections to hold the beams together.

Quasi-static modelling

1. Modelling of electrostatic force

The simplest model for electrostatic attraction force is based on the parallel-plate capacitor configuration, which assumes a pair of electrodes insulated by one or more dielectric materials. This model expresses the electrostatic force as [15]:

$$F_e = \frac{w\varepsilon_0 U^2 \varepsilon_{di} {\varepsilon_{in}}^2}{2(d_{in}\,\varepsilon_{di} + d_{di}\,\varepsilon_{in})^2} \tag{1}$$

where ε_0, ε_{di} and ε_{in} represent the permittivity of vacuum, the dielectric liquid, and the insulator, respectively. d_{in} and d_{di} denote the thicknesses of the insulator and liquid dielectric, w is the width of the electrode, and U is the applied voltage. This model is often employed for a simple estimation of electrostatic force. However, due to the interconnected mechanical and electrical responses of the system, the actual generated force changes dynamically [14]. We have developed an electromechanical model that accounts for the compression of PVC tape, coupled with large deformation beam theory [3]. Previous studies, such as Sîrbu, Ion-Dan, et al. [14] have shown that charge accumulation has a significant effect in electrostatic multilayer systems under DC voltage. Here, we extend our model by incorporating the effects of charge accumulation and compare materials to validate the model.

The model consists of three dielectric layers: two identical insulator layers and one liquid dielectric layer, as illustrated in Supplementary Fig. 8. The electrical conductivities of the insulator and the liquid dielectric are σ_{in} and σ_{di}, respectively. Due to the difference in conductivity, interface charges accumulate at the boundaries between the insulator and the liquid dielectric, leading to time-dependent changes in the electric fields of each

layer, denoted as E_{in} and E_{di}. The electrostatic force is derived from the Maxwell stress [16] of the liquid gap as

$$F_e = \frac{1}{2} w \varepsilon_0 \varepsilon_{di} E_{di}(t)^2 \tag{2}$$

Defining $J_{in,i}$ (i = 1, 2) and J_{di} as the current densities in each dielectric, based on the Gauss law, charge conservation at the interface can be expressed as

$$\begin{cases} J_{in,1} - J_{di} = -\dfrac{\partial}{\partial t}\left(\varepsilon_{in} E_{in,1} - \varepsilon_{di} E_{di}\right) \\ J_{di} - J_{in,2} = -\dfrac{\partial}{\partial t}\left(\varepsilon_{di} E_{di} - \varepsilon_{in} E_{in,2}\right) \end{cases} \tag{3}$$

According to Ohm's law, the equations can be expressed as

$$\begin{cases} \sigma_{in} E_{in,1} - \sigma_{di} E_{di} = -\dfrac{\partial}{\partial t}\left(\varepsilon_{in} E_{in,1} - \varepsilon_{di} E_{di}\right) \\ \sigma_{di} E_{di} - \sigma_{in} E_{in,2} = -\dfrac{\partial}{\partial t}\left(\varepsilon_{di} E_{di} - \varepsilon_{in} E_{in,2}\right) \end{cases} \tag{4}$$

The boundary condition is expressed as

$$U = E_{in,1} d_{in} + E_{di} d_{di} + E_{in,2} d_{in} \tag{5}$$

where U is the applied actuation voltage. From Supplementary Equations (4) and (5), we have

$$(\varepsilon_{in} d_{di} + 2\varepsilon_{di} d_{in}) \frac{\mathrm{d}E_{di}}{\mathrm{d}t} + (\sigma_{in} d_{di} + 2\sigma_{di} d_{in}) E_{di} = \sigma_{in} U + \varepsilon_{di} \frac{\mathrm{d}U}{\mathrm{d}t} \tag{6}$$

Under zero initial conditions, there is no current or charge accumulation at $t = 0$. Assuming DC voltage U_0 was applied, the electric field in the liquid dielectric can be solved as

$$E_{di}(t) = \frac{\sigma_{in}}{\sigma_{in} d_{di} + 2\sigma_{di} d_{in}} U_0 \left(1 - e^{-\frac{t}{\tau}}\right) + \frac{\varepsilon_{in}}{\varepsilon_{in} d_{di} + 2\varepsilon_{di} d_{in}} U_0 e^{-\frac{t}{\tau}}, \tag{7}$$

$$\tau = \frac{\varepsilon_{in} d_{di} + 2\varepsilon_{di} d_{in}}{\sigma_{in} d_{di} + 2\sigma_{di} d_{in}} \tag{8}$$

where τ is the time constant. $E_{di}(t)$ consist of two terms, an increment term related to conductivity and a decrement term related to dielectric constant. Thus, under DC voltage, the electrostatic force approaching

$$F_e = \frac{w\varepsilon_0 {U_0}^2 \varepsilon_{di} {\sigma_{in}}^2}{2(2d_{in}\,\sigma_{di} + d_{di}\,\sigma_{in})^2} \tag{9}$$

2. Modelling of large deformation beam

The actuator is composed of two identical beams, with the upper and lower beams experiencing forces of equal magnitude in opposite directions. The force loading point is located at the center of the beam in both the length and width directions, rendering the beam symmetrical about this center point and subject to identical force conditions. As illustrated in Supplementary Fig. 9a, when a liquid dielectric is present in the zipping area, the beam is divided into two regions: oil and non-oil regions. The length of the oil region is determined by the volume of liquid dielectric, extends as the liquid dielectric advances during zipping, and is considered to be zero when no voltage is applied.

Without limiting the generality, we focus on a quarter of the actuator in our parameterization (Supplementary Fig. 9b). We assume the curvature during static beam deformation to be tangential to the horizontal axis at both ends, effectively treating one end as fixed and the other as a movable clamp under vertical forces. The elliptic integral method is used to calculate the beam section without electrostatic force. A uniform beam with length L is shown in Supplementary Fig. 9b: Young's modulus of the material is E, and the moment of inertia of the section is $I = wh^3/12$, where w is the width and h is

the length of the beam. Through a large deflection analysis, three dimensionless equations can be derived:

$$\sqrt{\alpha} = F(\phi_2, k^2) - F(\phi_1, k^2) \tag{10}$$

$$\frac{b}{L} = -\frac{1}{\sqrt{\alpha}}[2E(\phi_2, k^2) - 2E(\phi_1, k^2) - F(\phi_2, k^2) + F(\phi_1, k^2)] \tag{11}$$

$$\frac{a}{L} = -\frac{2k}{\sqrt{\alpha}}(\cos\phi_2 - \cos\phi_1) \tag{12}$$

where a and b are projections of the beam on the X and Y axis, respectively. α is the dimensionless force which can be calculated by:

$$\alpha = \frac{RL^2}{EI} \tag{13}$$

where R is the vertical force acting on the ends of the beam. $F(\phi, k^2)$ and $E(\phi, k^2)$ are the incomplete elliptic integral of the first and second kind, respectively:

$$F(\phi, k^2) = \int_0^{\phi} \frac{d\delta}{\sqrt{1 - k^2 \sin\delta}}, E(\phi, k^2) = \int_0^{\phi} \sqrt{1 - k^2 \sin\delta}\, d\delta \tag{14}$$

The dimensionless parameter k is the modulus of the elliptic integral function and can vary between 0~1. In the large deflection problem, there is a rough correspondence between k and the magnitude of the force R. The variable ϕ is the amplitude of the elliptic integral, which continuously changes from ϕ_1 at the left end to ϕ_2 at the right end along the beam, and has the following relationship with the angle θ of the beam:

$$\sin\phi = \frac{1}{k}\cos\frac{\pi - 2\theta}{4} \tag{15}$$

In Supplementary Equation (15), ϕ_1 and ϕ_2 correspond to the angle at the left and right ends θ_1 and θ_2, respectively. In this case, θ_1 is constantly equal to 0. This approach includes the effects of both tip forces and moments. Supplementary Equations. (10)-(15) are the main equations of nonlinear beam analysis, requiring nonlinear numerical computation. The explicit expression of the moment at the left and right end is:

$$M_{1,2} = 2k\sqrt{EIR}\cos\phi_{1,2} \tag{16}$$

For any point on the beam, the coordinates after deformation can be expressed as:

$$\frac{y}{L} = -\frac{1}{\sqrt{\alpha}}[2E(\phi, k^2) - 2E(\phi_1, k^2) - F(\phi, k^2) + F(\phi_1, k^2)] \tag{17}$$

$$\frac{x}{L} = -\frac{2k}{\sqrt{\alpha}}(\cos\phi - \cos\phi_1) \tag{18}$$

The length from the left end to a point with magnitude ϕ is:

$$s = \sqrt{\frac{EI}{R}}[F(\phi, k^2) - F(\phi_1, k^2)] \tag{19}$$

In the active mode, the electrostatic force, primarily concentrated near the zipping point, is dominant in a small section and results in minimal deflection. This force is expressed as Supplementary Equation (9)We apply the Euler-Bernoulli beam theory to analyse the section containing oil. In the ith iteration, the relation between the bending moment M_{total} and the rotation angle θ in the jth micro-segment with length Δl is:

$$M_{total}(j, i) = EI\frac{\Delta\theta(j, i)}{\Delta l}, (i, j = 1, 2, \cdots, n) \tag{20}$$

During ith iteration, the ith segment is softened, and the rotation angle in that micro-segment can be calculated by:

$$\Delta\theta(i, i) = \frac{M_{total}(i, i)}{EI}\Delta l \tag{21}$$

The moment $M_{total}(i, i)$ acting on the ith segment is the sum of moment induced by distributed electrostatic force $M_q(i, i)$, moment induced by concentrated tip force $M_R(i, i)$ and moment at fixed boundary M_2, respectively, calculated as follows:

$$\begin{cases} M_{total}(i, i) = M_2 + M_R(i, i) + M_q(i, i) \\ M_R(i, i) = \sum_{j=1}^{i-1} R \cdot \Delta l \cdot \sin\left(-\frac{\pi}{2} - \theta(j, i-1)\right) \\ M_q(i, i) = \sum_{j=1}^{i-1}\left(q(i-j) \cdot \Delta l^2 \cdot \sum_{k=1}^{j} \sin\left(\frac{\pi}{2} - \theta(i-k, i-1)\right)\right) \end{cases} \tag{22}$$

The 1~ $(i-1)$th softening segment will undergo rotation due to the deformation of the

new softening segment ith. Therefore, the rotation angle of the segment $1\sim(i-1)$th is updated as follows:

$$\theta(j,i)=\theta(j,i-1)+\Delta\theta(i,i),\text{for } j=1,2,\dots,i-1. \tag{23}$$

After all segments are iterated (n iterations), the entire beam is softened. The deflection curve of the cantilever beam can be obtained, and the coordinates of the section jth can be calculated by:

$$\begin{cases} x(j)=\sum_{k=1}^{j}\Delta l\cos\big(\theta(n-k,n)\big) \\ y(j)=\sum_{k=1}^{j}\Delta l\sin\big(\theta(n-k,n)\big) \end{cases} \tag{24}$$

In simulation, the actuator extension is fixed, and the contractile force is calculated under different applied voltages. A comprehensive coupling method and computational process are detailed in reference [3].

3. Modelling of ERF beam

To analyse the stiffness variation characteristics of ESRA, a basic beam element consisting of two flexible electrodes with a power supply voltage of V was studied, as shown in Supplementary Fig. 10a. The electrode thickness is d_e. Both sides of one of the electrodes are covered with an insulating film of thickness d_{in}. A layer of ERF film with a thickness of d_f is filled between the electrodes. Defining $h=d_f+d_{in}+2d_e$ (Supplementary Fig. 10b), the beam structure can be equivalent to a sandwich I-beam structure when the thickness of the face sheets (electrodes) is smaller than that of the overall sandwich thickness ($d_e \ll h$). In a sandwich beam, the face sheet mainly affects the bending stiffness K_b, while the core mainly affects the shear stiffness K_s [1]:

$$\begin{cases} K_b \approx \dfrac{w\left(h^3 - d_f^3\right)}{12} E_e \\ K_s \approx \dfrac{\left(d_f + d_e\right)^2}{d_f} (w_f G_f + w_s G_s) \end{cases} \tag{25}$$

where E_e is the Young's modulus of the electrode material. w, w_f and w_s are the width of the electrodes, ERF, and spacer, respectively. G_s is the shear stiffness of the spacer, expressed as $G_s = E_s/2(1 + \mu_s)$ with Young's modulus E_s and Poisson's ratio μ_s of the spacer, respectively. G_f is the shear modulus of the ERF, which is the ratio of shear stress τ and shear strain rate $\dot{\gamma}$. The shear stress of ERF in an electric field can be described by the Bingham plastic model [2]:

$$\begin{cases} \tau(\dot{\gamma}) = \tau_0(E_f) + \mu\dot{\gamma}, \text{ at } \tau > \tau_0 \\ \dot{\gamma} = 0, \text{ at } \tau < \tau_0 \end{cases} \tag{26}$$

where $\tau_0(E_f)$ is the yield shear stress under an electric field E_f, and μ is the dynamic viscosity of the ERF. According to Supplementary Equations (25) and (26), under shear stress, a displacement occurs after the yield stress has been overcome. Below the yield stress, ERF behaves like a solid, and above the yield stress it acts as a very high-viscosity liquid. The electric fields in the insulator E_{in} and ERF layers E_f are related as follows:

$$V = E_{in} d_{in} + E_f d_f \tag{27}$$

Assuming that the electric field in the absence of a medium is E_0, the electric field in the medium can be represented as:

$$E_i = \frac{\varepsilon_0 E_0}{\varepsilon_i} \tag{28}$$

where $i = in, f$. ε_0 and ε_i are the permittivity of vacuum and medium, respectively. Combing Supplementary Equations (27) and (28), the electric field in the insulator and ERF layer can be calculated as:

$$E_f = \frac{\varepsilon_{in} V}{\varepsilon_f d_{in} + \varepsilon_{in} d_f}, E_{in} = \frac{\varepsilon_f V}{\varepsilon_f d_{in} + \varepsilon_{in} d_f} \tag{29}$$

With an applied voltage, the induced electric field allows ERF to withstand significantly higher shear stresses and exhibit greater bending stiffness. In the case of three-point bending, the deflection at a beam's mid-point may be given as the sum of the deflections due to bending of the face sheets and shear of the core:

$$y = F\left(\frac{L^3}{48K_b} + \frac{L}{4K_s}\right) \tag{30}$$

where L is length of the beam and F the force at the mid-point.

Owing to the slender beam assumption in the large deformation beam model, it does not account for the beam's shear force effect. This leads to a decline in result precision when considering the shear modulus of the ERF layer in ESRA. However, considering shear effect, the equivalent flexural stiffness is approximated as

$$(EI)_{\text{eff}} = \frac{K_b}{1 + 12\frac{K_b}{K_s L^2}} \tag{31}$$

Thus, the normalized equivalent flexural stiffness is $(EI)_{\text{eff}}/w$.

4. Validation

Simulation and validation of the electromechanical model considering charge accumulation

The proposed model, featuring electro-ribbon actuators, was simulated and its results compared with those obtained from an isometric experimental setup as shown in

Supplementary Fig. 11. Prior to voltage application, the actuator was extended to predetermined positions and the contractile force was recorded. The actuator's midpoint is secured; one segment is connected to a load cell (DBCR-10N-002–000, Applied Measurements Ltd., UK), and the opposite end is mounted to a bracket on a manual fine-tuning platform. Before testing, a drop of silicone oil (50 cSt viscosity, Sigma-Aldrich, USA) is added at each zipping point to ensure consistency. The contractile force measurements are taken when the force data stabilizes.

Both simulated and tested actuators have a beam length of 90 mm and a width of 12.7 mm. The contractile forces were evaluated at fixed extensions of 8 mm and 18 mm. Three types of insulator materials were tested to validate the simulation results: PVC tape (AT7, Advanced Tape, UK), polyimide (PI) film by DuPont™ Kapton, and biopolyester (BP) film (Naturabiomat GmbH, Austria) [14]. The thickness d, dielectric constant ε, electrical conductivity σ for each material are detailed in Supplementary Table 3.

Supplementary Fig. 12 illustrates the contractile forces under various actuation voltages, comparing isometric computational and experimental results. Overall, the predicted force values align well with the experimental data, demonstrating an increase in force in the inactive case (0 kV) and a reduction in force as the extension increases under a constant voltage in the active case. For PVC and BP films, the contractile force significantly rises with voltage application, showing comparable performance attributed to their higher conductivity compared to the liquid dielectric. However, the BP film tends to break down at voltages around 5 kV. The predicted values at high voltages slightly overestimate the

actuator's performance, likely due to imprecise edge constraints which become more evident under significant contractile forces. When using PI, the contractile forces at a steady state closely resemble those in the inactive state, indicating a negligible increase upon voltage application. Despite PI's similar dielectric constant and the thinnest profile among the tested materials, its performance is the poorest, failing to facilitate gradual zipping for large deformations.

Validation of ERF beam model

To determine the value of yield shear stress τ_0, we refer to the empirical relationship between yield stress and electric field density outlined in reference [4], which employs the same electrorheological fluid (ERF) material as our study. The simulated sandwich structure consists of 12.7 mm wide and 30 μm thick electrodes and have a Young's Modulus of 190 GPa (matching the electrode properties used in actuators). The spacer has a Young's modulus of 459 kPa and a Poisson's ratio of 0.499. The spacer width was 1.8 mm. The thickness of ERF was 0.5 mm and the width was 10.9 mm. Vacuum permittivity was 8.85 pF/m, insulator relative permittivity was 4.62 and insulator thickness was 130 μm (matching the insulator properties used in actuators). Supplementary Fig. 13 shows the rheological behavior of ERF in an ERF beam. The electric field density in ERF layer increases with the applied voltage. Corresponding to the electric field density, the shear yield stress increases from 0 to approximately 50 kPa. Subsequently, we calculate the displacement at the central point of a sandwich beam subjected to three-point bending, as depicted in Supplementary Fig. 10a (simulation

result is shown in Fig. 2b). The simulated beam here is 48 mm long and experiences a load of 0.1176 N.

Simulation and Validation of ESRA

In our simulation exploring the force-extension characteristics of the actuator, we employed a beam length of 80 mm, maintaining consistency in material properties with those used in the ERF beam simulation. Fig. 2c presents the results, showcasing the simulated force-extension characteristics of the actuator across a range of normalized equivalent bending stiffness values. The assumption of uniform bending stiffness may not fully apply in experimental conditions, which could contribute to discrepancies between simulated and experimental data.

The theoretical contractile force of the actuator was calculated using its dimensional parameters in an isometric test and subsequently compared to experimental data. Fig. 4g presents the comparison of contractile force under conditions of 0 kV and 6 kV stiffening voltage in the passive state (0 kV actuation voltage). Fig. 4h shows the comparison under an actuation voltage of 7 kV. Beyond the idealized assumption of uniform bending stiffness, edge effects in the electric field may also influence the result. Variations in electric field strength along the ribbons may affect the bending stiffness distribution, contributing to observed differences between simulation and experimental data in Figures 4g and 4h.

Analysis of equivalent dynamic system of ESRA

The force-deformation characteristics of electrostatic actuators exhibit significant nonlinearity in both inactive and active states. These characteristics in ERA and ESRA closely resemble those found in biological muscles. To capture the fundamental dynamics of ESRA, a muscle model is employed [5]. The three-element muscle model [6], originally developed to simulate skeletal muscle contraction and stimulation, serves as the foundation for the dynamic characterization of ESRA. This model simplifies ESRA into a parallel configuration comprising a nonlinear spring element, a nonlinear damping element, and a contractile element, as depicted in Supplementary Fig. 14a. The contractile element is an addition to the conventional mass-spring-damper system, representing the internal contractile force generated by electrostatic forces. According to Newton's Second Law, the dynamic equilibrium equation is expressed as:

$$m\ddot{x} = \mathbf{F_{ce}} + \mathbf{F_i} + \mathbf{F_b} \tag{32}$$

where $\mathbf{F_{ce}}$ is the contractile force produced by the contractile element, $\mathbf{F_i}$ is the internal force produced by spring and damper, and $\mathbf{F_b}$ represents the body force, such as gravity. Due to the nonlinear nature of the force-tensile properties, the introduction of independent stiffness changes substantially increases the system's complexity. Here, a reduced-order method is employed, utilizing small amplitude characterization at the equilibrium position for linearization approximation. The small amplitude linearization model is illustrated in Supplementary Fig. 14b, where the contractile element is replaced by a spring with an elastic coefficient of k_{ES}, and the nonlinear spring and damping elements are substituted by the local effective stiffness $k_{mat.}$ and effective damping c. The reduction is expressed as:

$$\mathbf{F_{ce}} + \mathbf{F_i} = c(V_{stiff.})\dot{x} + k_{ES}(V_{act.}, V_{stiff.})x + k_{mat.}(V_{stiff.})x \tag{33}$$

where $k_{mat.}(V_{stiff.})$ represents the material and structural stiffness, which varies with the applied stiffening voltage; $c(V_{stiff.})$ is the damping coefficient that varies with the applied stiffening voltage; and $k_{ES}(V_{act.}, V_{stiff.})$ is equivalent stiffness of the electrostatic force, dependent on both applied actuation and stiffening voltages. The body force is only due to gravity:

$$\mathbf{F_b} = m\mathbf{g} \tag{34}$$

At the equilibrium state, the spring force balances with gravity, leading to the dynamic equation:

$$m\ddot{x} + c(V_{stiff.})\dot{x} + \left(k_{ES}(V_{act.}, V_{stiff.}) + k_{mat.}(V_{stiff.})\right)x = 0 \tag{35}$$

In the passive state, where ESRA operates without an actuation voltage, $k_{ES}\left(0, V_{stiff.}\right) = 0$. In this case the equation simplifies to:

$$m\ddot{x} + c(V_{stiff.})\dot{x} + k_{mat.}(V_{stiff.})x = 0 \tag{36}$$

Analysis of one-dimensional simplification of the agonist–antagonist system

In the agonist-antagonist system, the vertical arrangement is configured as ESRA-platform-ERA from top to bottom, as shown in Supplementary Fig. 15. To model the ESRA, we employ the previously described spring-mass-damper system. The ERA, characterized by relatively high stiffness and negligible damping compared to the ESRA, is modelled using a contraction unit and a spring unit. These two components are connected by a point mass, with both the upper and lower springs initially extended. The fixed upper and lower ends ensure that the total system length remains constant.

When the mass is displaced by a distance x from its equilibrium position, the upper spring elongates by x, while the lower spring shortens by the same distance. The resulting equation of motion is given by:

$$m\ddot{x} = -(k_{ES} + k_{mat.} + k_{ERA})x - c\dot{x} \tag{37}$$

the contractile force of the ERA is treated as an external excitation force, leading to the dynamic equation:

$$m\ddot{x} + c\dot{x} + (k_{ES} + k_{mat.} + k_{ERA})x = F \tag{38}$$

Thus, the effective stiffness k_{eff} and damping c_{eff} of the system is expressed as:

$$k_{eff} = k_{ES} + k_{mat.} + k_{ERA}, \;\; c_{eff} = c \tag{39}$$

Analysis of actuation system in series

A model comprising two ESRA in series is illustrated in Supplementary Fig. 16a. In this model, F is an excitation force while x and x_m denote the displacements of load M and the middle point between the two actuators, respectively. The system's equivalent variable stiffness and damping configuration is represented in Supplementary Fig. 16b. Here k' and c' are equivalent stiffness and damping coefficient, respectively. The equation of motion for the system in Supplementary Fig. 16a are presented as follows:

$$\begin{bmatrix} 0 & 0 \\ 0 & M \end{bmatrix} \begin{Bmatrix} \ddot{x}_m \\ \ddot{x} \end{Bmatrix} + \begin{bmatrix} c_1 + c_2 & -c_2 \\ -c_2 & c_2 \end{bmatrix} \begin{Bmatrix} \dot{x}_m \\ \dot{x} \end{Bmatrix} + \begin{bmatrix} k_1 + k_2 & -k_2 \\ -k_2 & k_2 \end{bmatrix} \begin{Bmatrix} x_m \\ x \end{Bmatrix} = \begin{Bmatrix} 0 \\ F_0 e^{i\omega t} \end{Bmatrix} \tag{40}$$

where $F = F_0 e^{i\omega t}$ and ω is the excitation frequency. Simplifying the analysis, we neglect the effect of c_2. The compliance is expressed as

$$\frac{X}{F_0} = \frac{-1}{-m\omega^2 + k_1 - \left(\frac{k_1^2(k_1 + k_2)}{(k_1 + k_2)^2} + c_2^2\omega^2\right) + \mathrm{i}\left(\frac{k_1^2 c_2}{(k_1 + k_2)^2} + c_2^2\omega^2\right)\omega} \tag{41}$$

The corresponding transfer function for the equivalent model is

$$\frac{X}{F_0} = \frac{-1}{-m\omega^2 + k' + ic'\omega} \tag{42}$$

Upon comparing Supplementary Equation (41) with Supplementary Equation (42), the equivalent stiffness and damping coefficients are derived as follows

$$k' = k_2 - \frac{k_2^2(k_1 + k_2)}{(k_1 + k_2)^2 + c_1^2\omega^2} \tag{43}$$

$$c' = \frac{k_2^2 c_1}{(k_1 + k_2)^2 + c_1^2\omega^2} \tag{44}$$

Consequently, controlling k_1 and c_1 enables an increase in equivalent stiffness without a corresponding rise in damping coefficient. Conversely, adjusting k_2 and c_2 results in an increase in both equivalent stiffness and damping coefficients.

Supplementary Figures

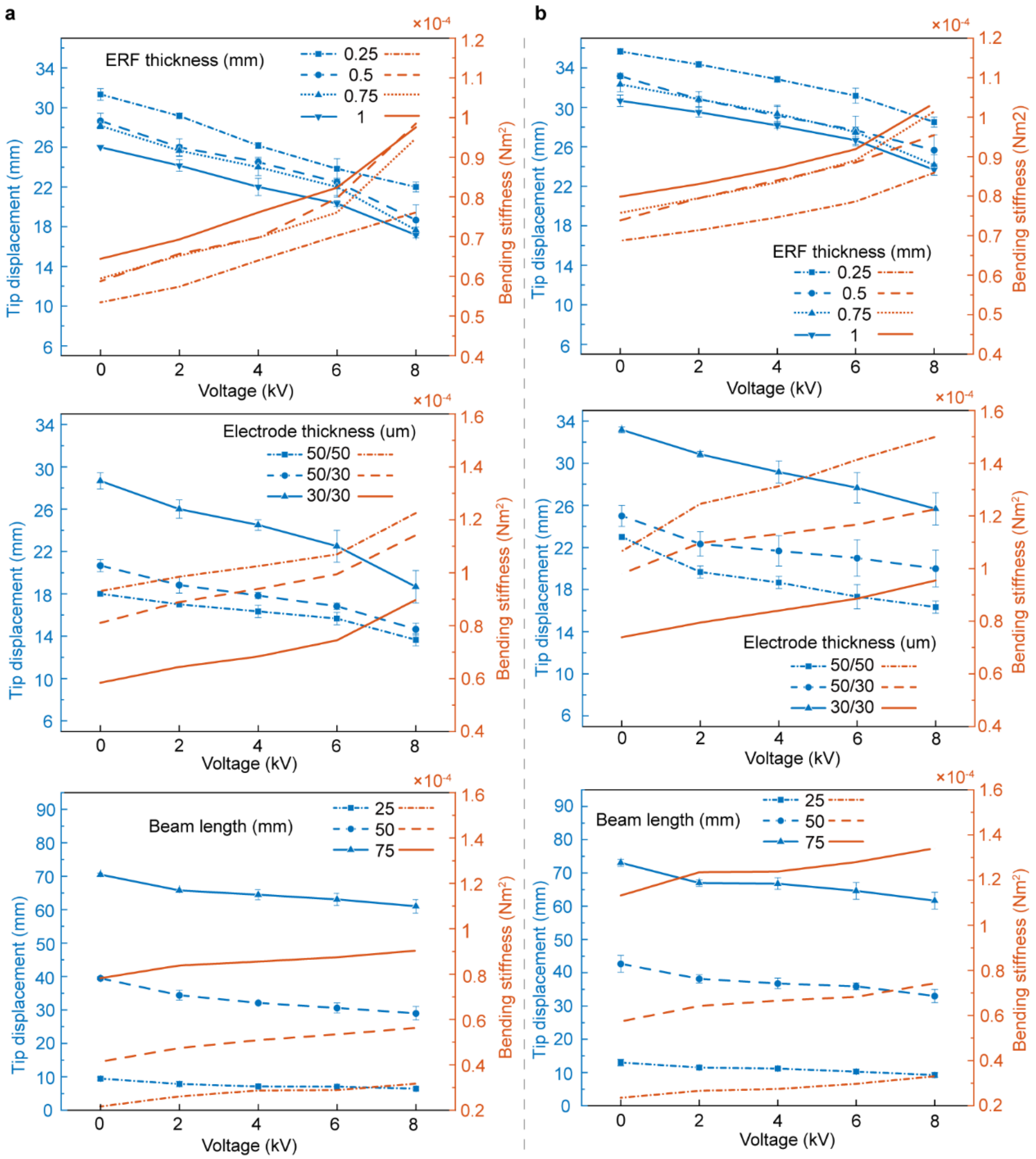

Supplementary Fig. 1: Comparison of deflection and bending stiffness of ERF beam.

ERF beams with different electrode thickness, ERF layer thickness, and beam length are applied with various stiffening voltages. **a** and **b** depict the responses under 6 g and 4 g loads, respectively. The blue line represents beam tip displacement (left axis), and the red line represents bending stiffness (right axis). The error bars indicate standard deviation between 3 trials.

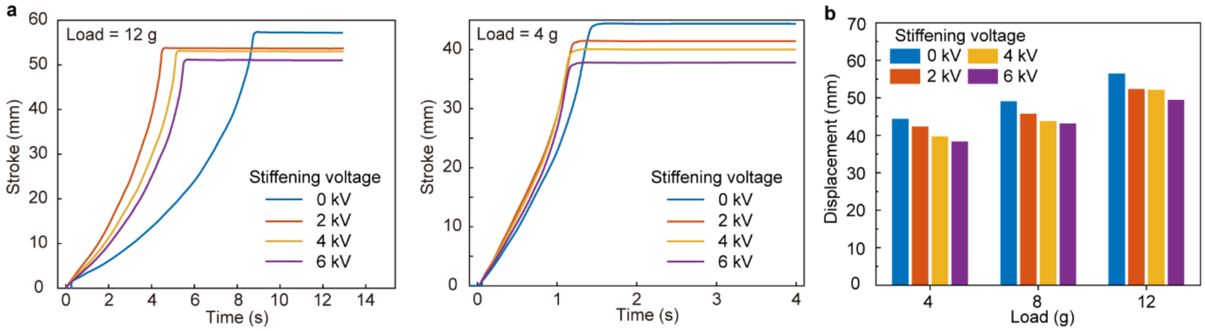

Supplementary Fig. 2: Isotonic test and comparison of static extension of an actuator.

a Comparison of stroke of an actuator with 30/30 um electrodes, 0.5 mm ERF thickness and 7kV actuation voltage, lifting loads of 4 and 12 -g under four different stiffening voltages. **b** Extension response of a ESRA with 30/30 μm electrodes and 0.5 mm ERF layers under different loading conditions. The actuator is subjected to loads of 4, 8, and 12 g, respectively. Prior to loading, four distinct stiffening voltages are applied to assess their impact on the actuator’s extension behavior.

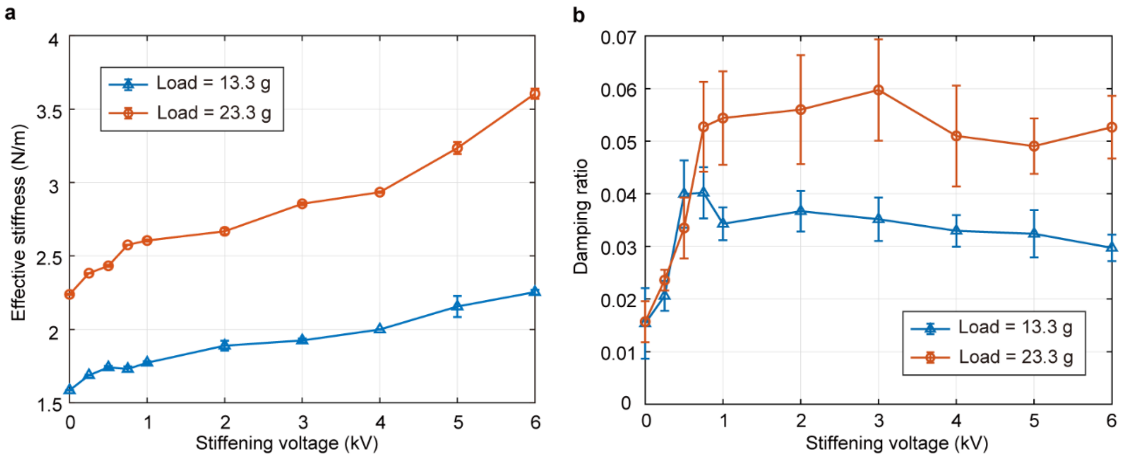

Supplementary Fig. 3: Effective stiffness and damping ratio from free response

a Effective stiffness at various stiffening voltages under loads of 13.3 g and 23.3 g **b** Damping ratio at various stiffening voltages under loads of 13.3 g and 23.3 g. The error bars indicate standard deviation between 3 trials.

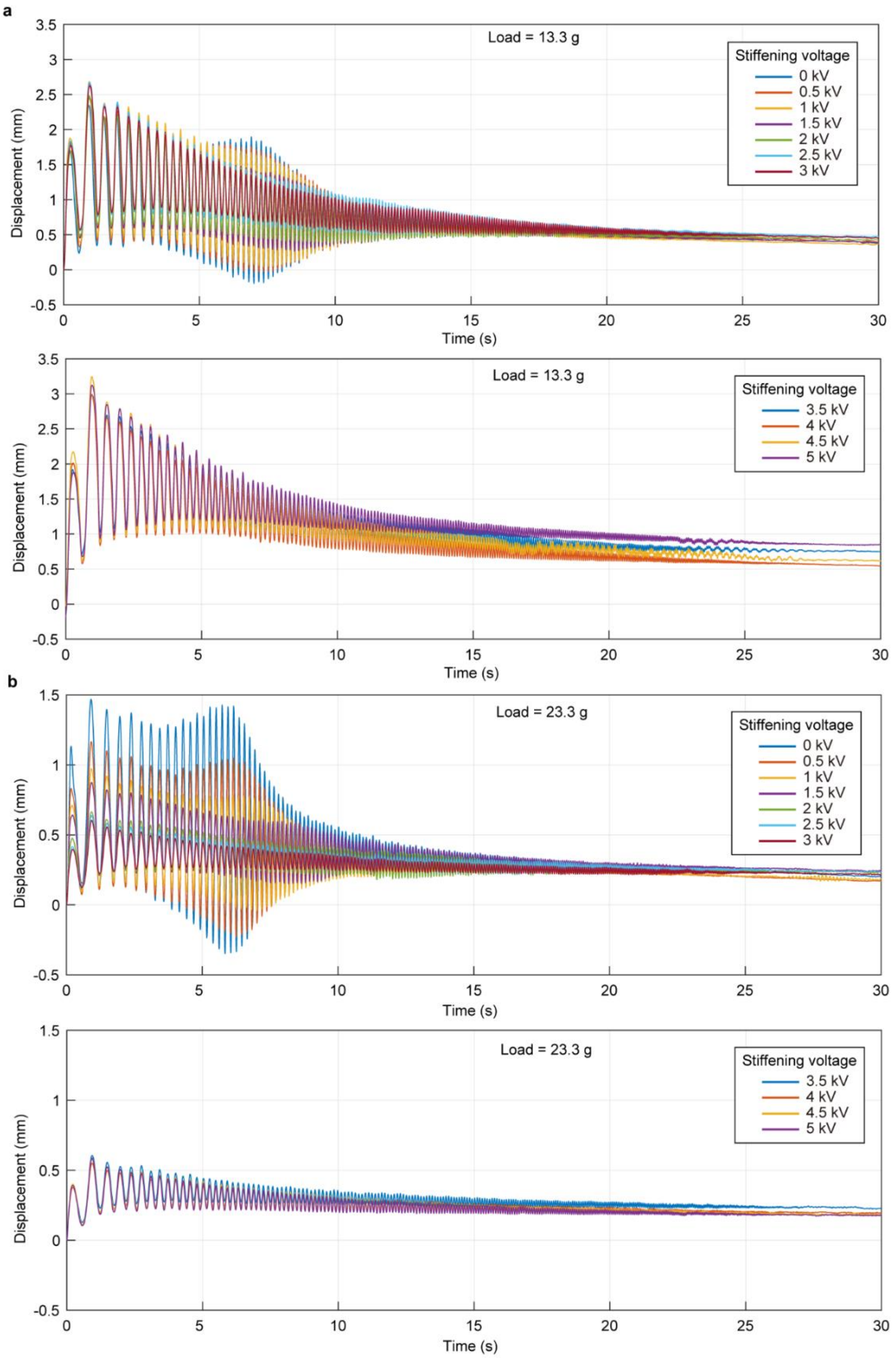

Supplementary Fig. 4: Time response to a chirp signal excitation.

a Time response at various stiffening voltages under loads of 13.3 g. **b** Time response at various stiffening voltages under loads of 23.3 g.

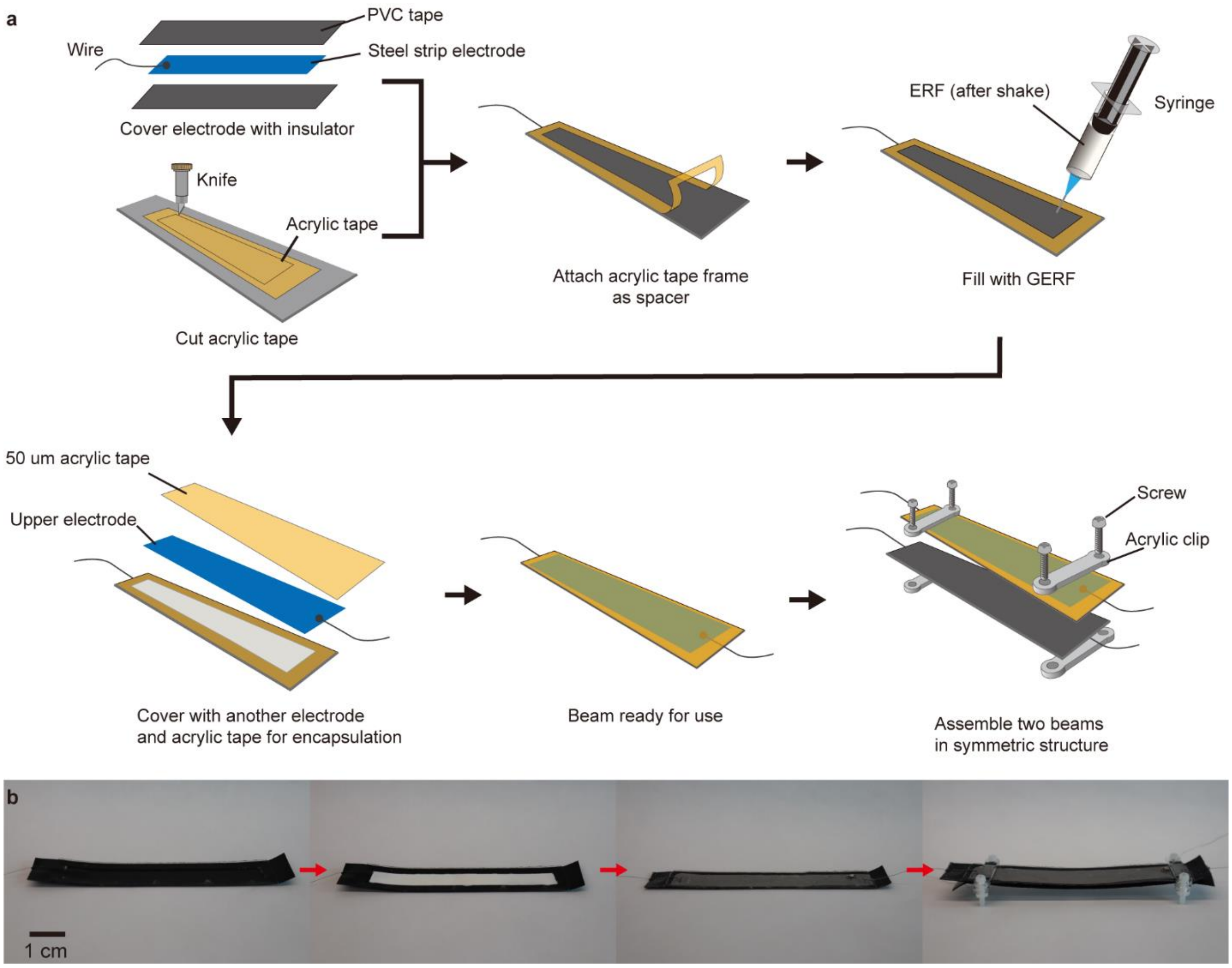

Supplementary Fig. 5: Fabrication procedure of variable stiffness beams and actuators.
a Representative manufacturing steps for ESRA. **b** photographs showcasing variable stiffness beam and actuator.

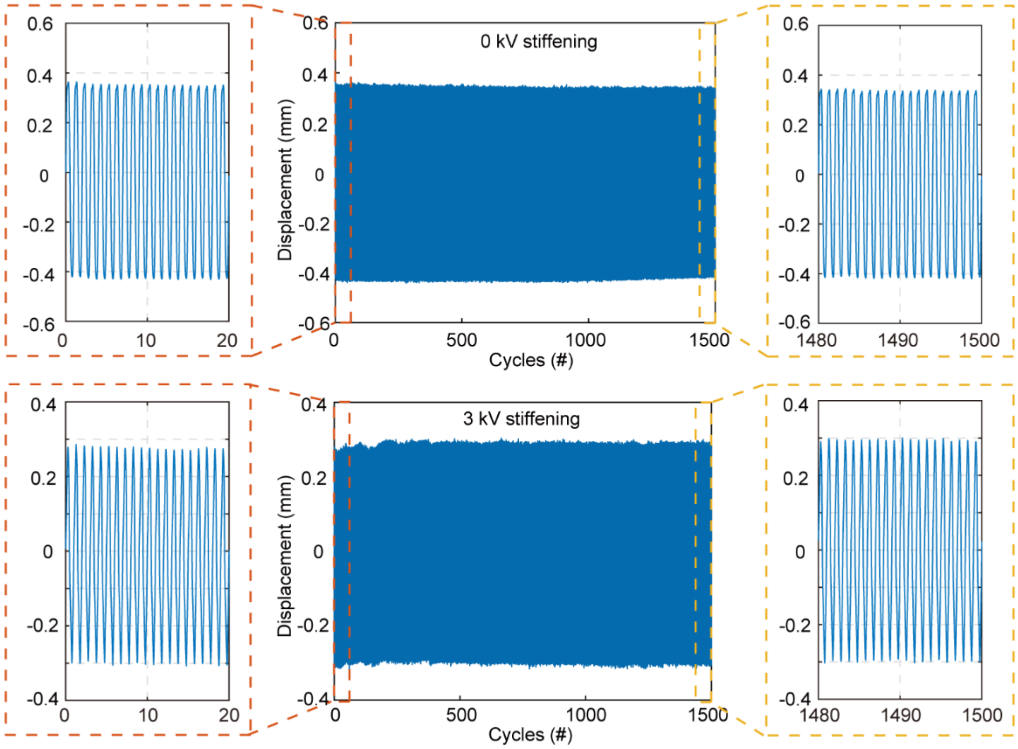

Supplementary Fig. 6: Durability test

Durability test of ESRA over 1500 actuation cycles, showing displacement lifting 17 g with a bipolar signal of 5 kV at 2 Hz with and without stiffening. The red and yellow insets show results at the beginning and end of the durability test, respectively.

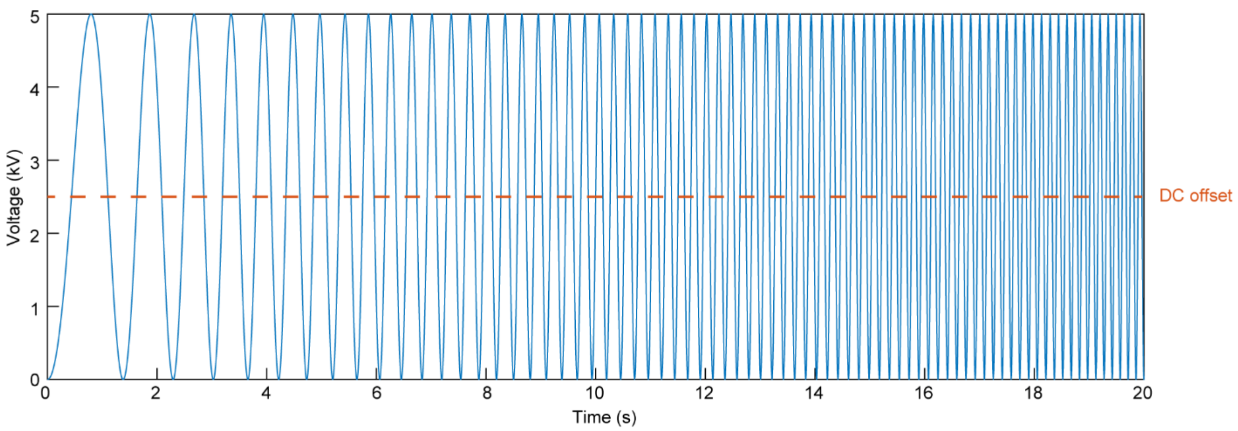

Supplementary Fig. 7: A typical sinusoidal frequency chirp signal used for dynamic characterisation.

The signal consists of two parts: a 2.5 kV DC offset voltage and an AC voltage with the amplitude of 2.5 kV. The frequency is increased continuously from 0.5 to 20 Hz in 60 seconds, the figure shows the first 20 seconds.

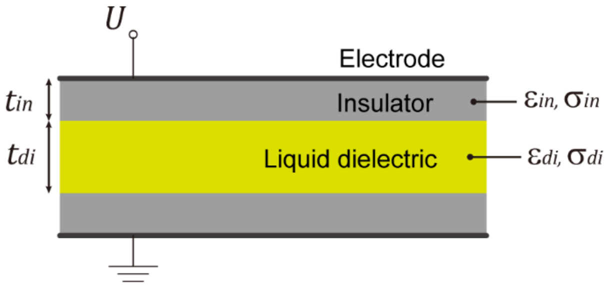

Supplementary Fig. 8: Multilayer parallel capacitor for electrostatic contraction.
Schematic representation of a multilayer parallel capacitor structure used for electrostatic contraction. The capacitor consists of an insulator layer with thickness t_{in}, relative permittivity ε_{in}, and conductivity σ_{in}, and a liquid dielectric layer with thickness t_{di}, relative permittivity ε_{di}, and conductivity σ_{di}. An applied voltage U across the electrodes generates electrostatic forces within the layers, enabling contraction.

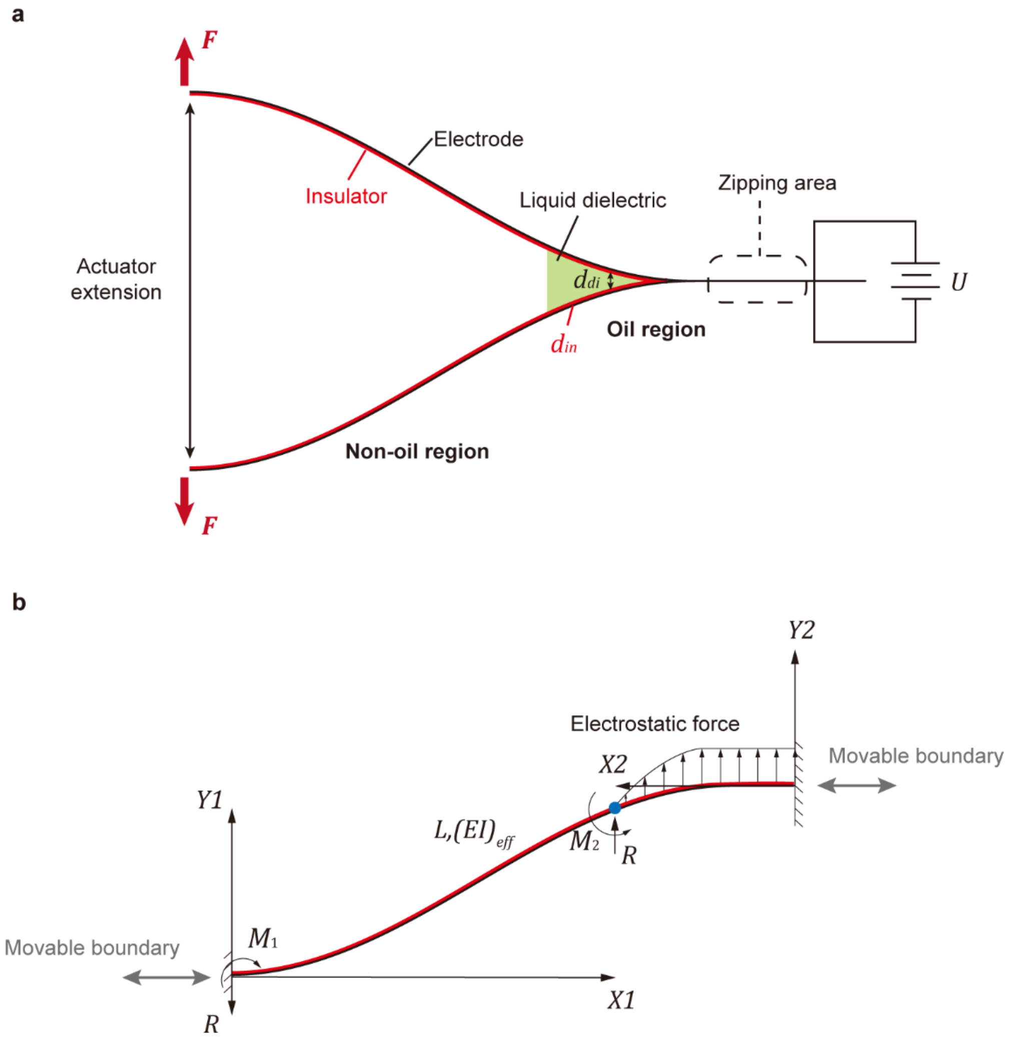

Supplementary Fig. 9: Electromechanical model of the actuator.

a Model and mechanism of half of the zipping structure. The oil region is the beam section covered by liquid dielectric with distance d_{di} and insulator with thickness d_{in}; the non-oil region is the beam section without liquid dielectric. An applied voltage U creates an electrostatic force in the zipping area, while forces F act at the ends of the actuator. **b** Parameterization of the actuator shape, a quarter of the actuator is regarded as a beam with movable clamped at two ends. The length and effective stiffness of the beam are represented as L and EI_{eff}, respectively. The non-oil section experiences a tip force R and moment M_1, while the oil section is subjected to a tip force R, a tip moment M_2, and distributed electrostatic forces. The blue point represents the boundary connecting point between two sections.

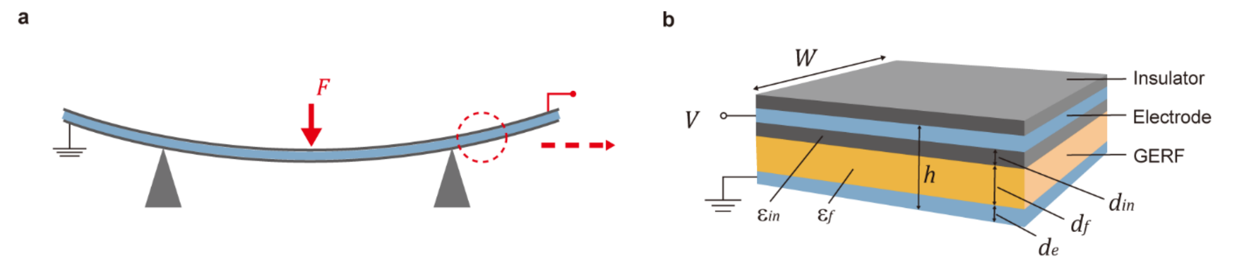

Supplementary Fig. 10: Electrorheological Fluid (ERF) beam model.

a Schematic diagram three-point bending of a basic ERF beam. The applied force F at the center induces deformation, illustrating the beam's bending behaviour under load. **b** Sandwich beam structure of the ERF beam and basic parameter. The structure includes an insulator layer, electrode layers, and an ERF layer. The thicknesses of the insulator, electrode, and ERF layers are denoted as d_{in}, d_{e}, and d_f, respectively. The width of the beam is W, and the total thickness of the electrode covered layer is h. The relative permittivity of the insulator and ERF layers are represented as ε_{in} and ε_f. An applied voltage V generates an electric field.

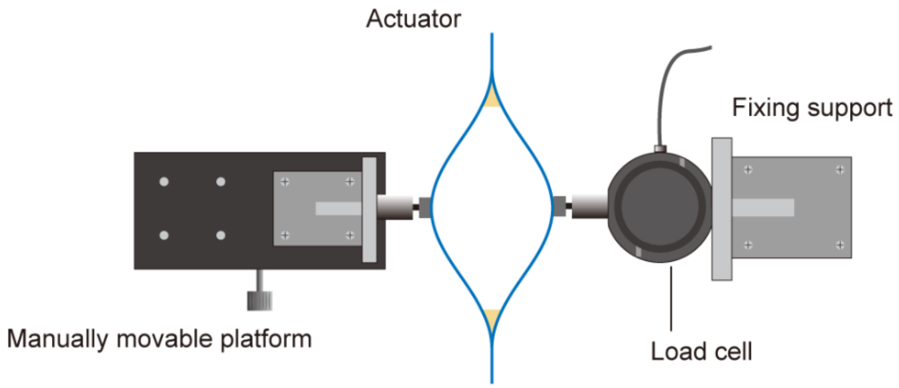

Supplementary Fig. 11: The isometric test setup to measure force at a certain extension.

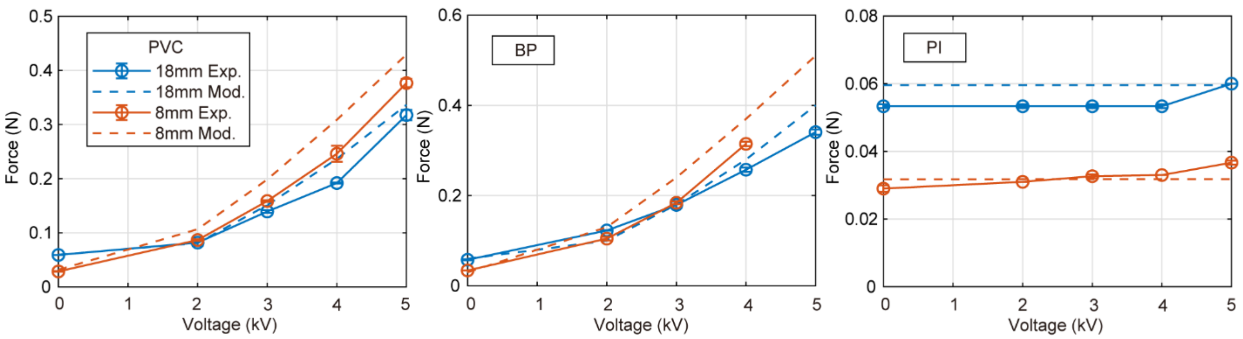

Supplementary Fig. 12: Comparison of model and experimental results.

The results for PVC, BP, and PI materials under various voltages are compared, where dashed lines represent the modelled results and solid lines depict the experimental outcomes. Blue lines indicate the contractile force at 8 mm extension, while red lines illustrate the force at 18 mm extension. The error bars indicate standard deviation between 3 trials.

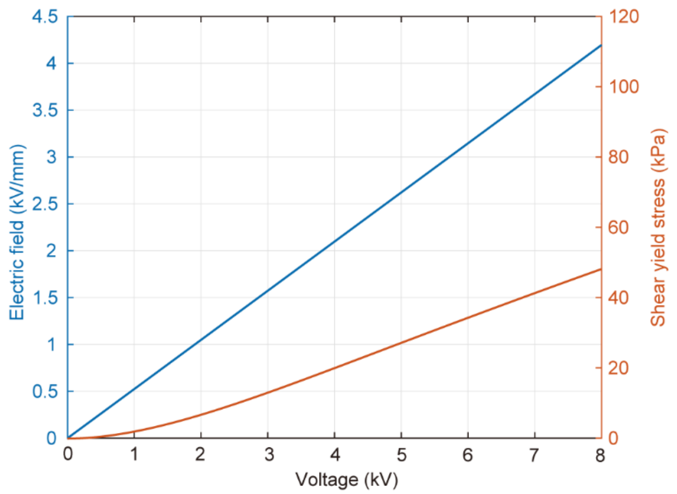

Supplementary Fig. 13: Rheological behavior of ERF.

The left blue line illustrates the correlation between the electric field in the ERF layer and the voltages applied to the electrodes. The right red line demonstrates the variation of shear yield stress in response to different electrode voltages. The underlying relationship between shear yield stress and electric field strength is fitting data from empirical data [4]. The depicted yield stress values (red line) are then obtained by fitting data according to the calculated electric field strength (blue line).

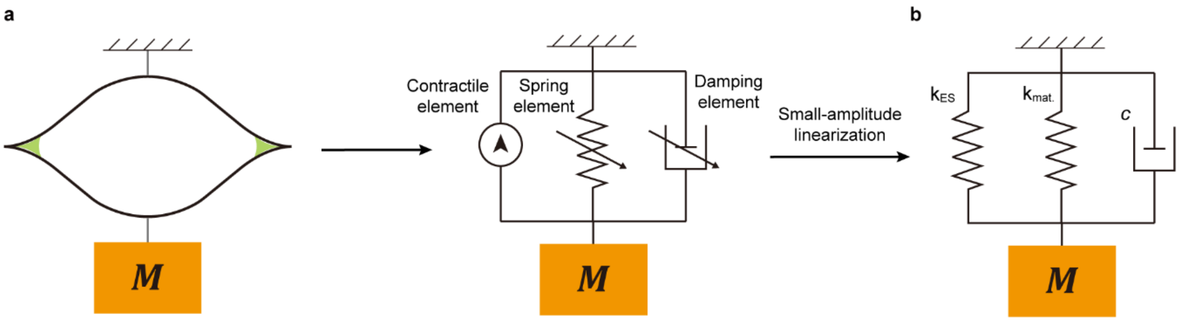

Supplementary Fig. 14: Equivalent dynamic model of the Electro-Stiffen Ribbon Actuator (ESRA).

a Schematic drawing of the ESRA with a load, and the equivalent three-element model consisting of a contractile element, a spring element, and a damping element. **b** Simplified spring-damper-mass model after linearization approximation, where k_{ES} and $k_{mat.}$ are the spring coefficients of the electrostatic force and material, respectively, and c is the damping coefficient of the system.

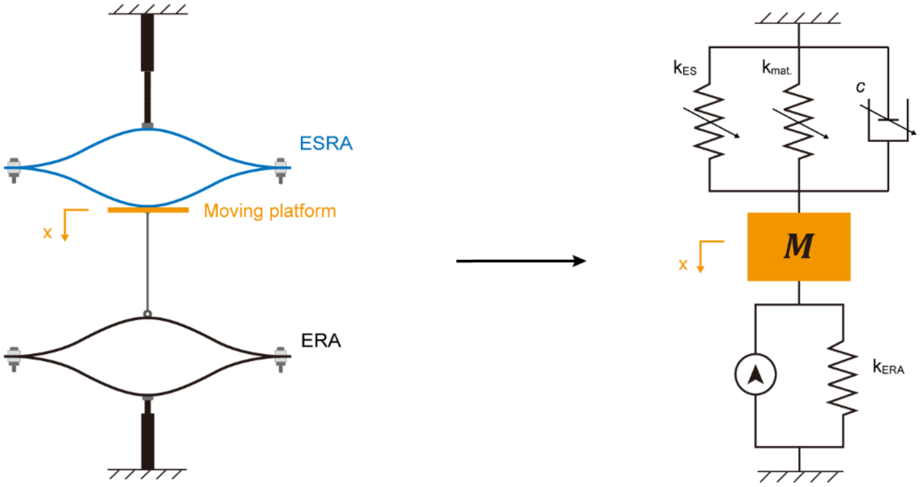

Supplementary Fig. 15: One-dimensional simplification of the agonist–antagonist system

Schematic drawing of the agonist–antagonist test and the equivalent system model. The vertical arrangement is configured as ESRA-platform-ERA from top to bottom. The spring-damper system represents the behaviour of the Electro-Stiffen Ribbon Actuator (ESRA), while a contractile element and spring element describe the Electro-Ribbon Actuator (ERA). k_{ES}, $k_{mat.}$ and k_{ERA} are the spring coefficients of the ESRA's electrostatic force, ESRA's material and ERA, respectively, and c is the damping coefficient of the ESRA.

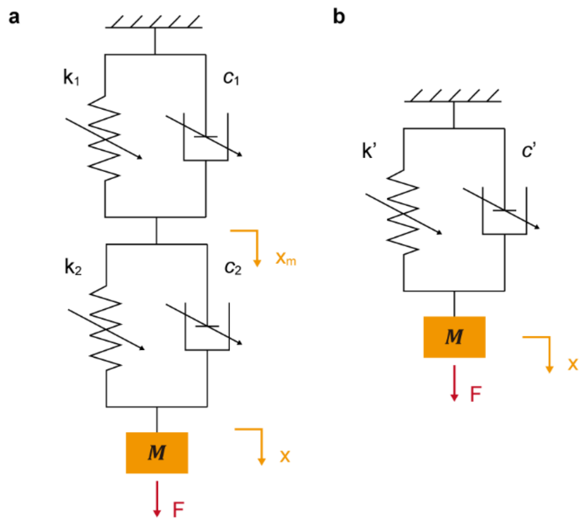

Supplementary Fig. 16: Mechanical configuration of two Electro-Stiffened Ribbon Actuator (ESRA) in series

a The system is configured with two spring-damper elements arranged in series, with stiffness values k_1 and k_2, and damping coefficients c_1 and c_2. The mass M is connected to the second spring-damper system, with displacement denoted as x, while the intermediate node displacement is represented as x_m. An external force F acts on the mass. **b** Equivalent variable stiffness k' and damping c' model.

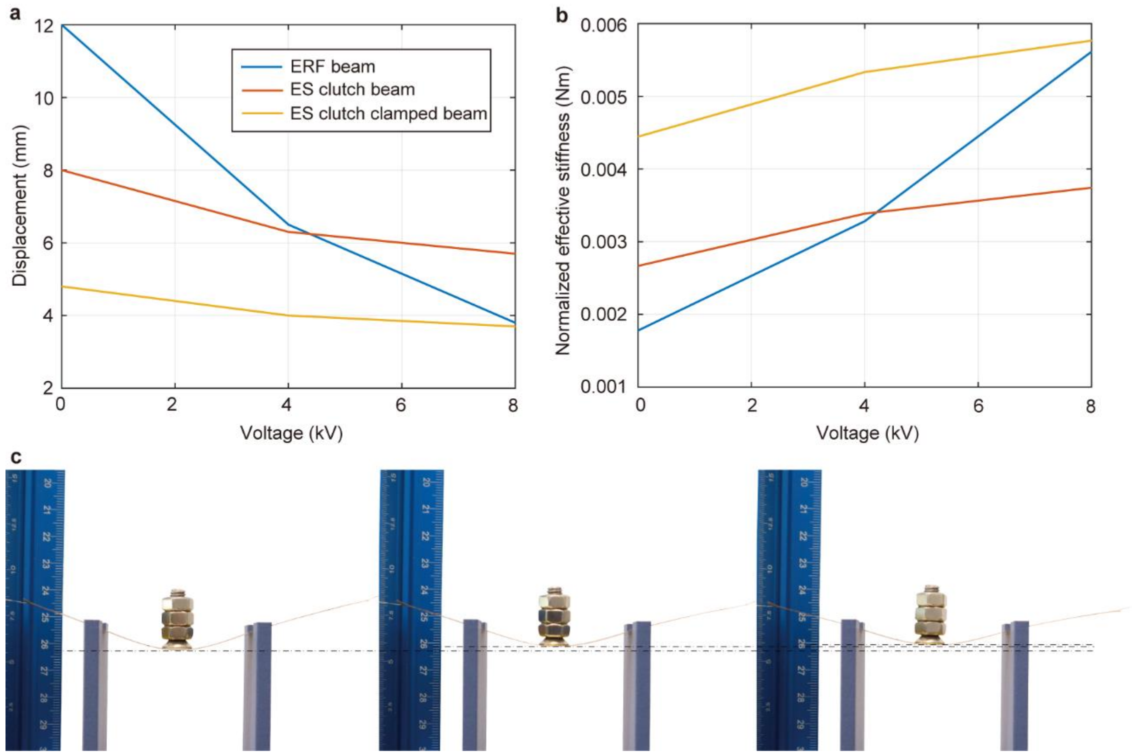

Supplementary Fig. 17: Comparison of three-point bending test result.

a Displacement results from three-point bending tests on an Electro-Rheological Fluid (ERF) beam, an Electrostatic (ES) clutch beam, and an ES clutch beam with a clamp. The ES clutch beam consists of two 30 µm steel electrodes and two layers of 50 µm Kapton tape (DuPont) positioned at the center. The gap between two supporting points is 48 mm. **b** Normalized effective stiffness derived from the displacement results in **a**. **c** Three-point bending test of ES beam.

Supplementary Table 1: Comparison of key parameters between this work and different soft actuators.

References	Actuation	VS/VD mechanism	Control	Response time (s)	Power consumption (W)	VS/VD for actuation enhancing*	Stiffness variation	Damping ratio variation
This work	ES	ER	Electric	< 0.01 [7]	< 0.025 [7]	Yes	~ 8 ×	~ 3 ×
8	PAM	LJ	Vacuum	> 3.9 [9]	/	Yes	~ 6 ×-	/
13	PAM	PJ	Vacuum	> 2.5	/	No	~ 6 ×	/
10	DE	DE	Electric	~ 0.01 [11]	< 0.01 [11]	No	~ 3.5 ×-	/
12	DE	LMPA	Electric	30 – 60	≥ 1	No	~ 90 ×	/
17	PAM	PJ	Vacuum	> 1	/	Yes	< 10 × [18]	~ 1.8 ×-
19	DE	DE	Electric	~ 0.01 [11]	< 0.01 [11]	No	4.2 ×	< 2 ×

VS (variable stiffness), VD (variable damping), ES (electrostatic), ER (electro rheology), PAM (pneumatic artificial muscle), LJ (layer jamming), PJ (particle jamming), DE (dielectric elastomer), LMPA (low melting point alloy).

* An increase in stiffness can enhance the actuation response while also improving the passive payload capacity.

- These data are not directly given by the corresponding references, and we read original data from graphs to calculate the properties.

Supplementary Table 2: Comparison of key parameters between series ESRA and other variable stiffness and damping systems.

References	VS/VD mechanism	Elements	Stiffness variation	Damping ratio variation	Actuation capability
This work	ER	2	2.2 ×	2.6 ×	Yes
20	MR	4	2.8 ×	3.6 ×	No
21	MR	3	2.7 ×	2.3 ×	No

VS (variable stiffness), VD (variable damping), ER (electro rheology), MR (magneto rheology)

Supplementary Table 3: Material properties of dielectric materials.

Dielectric material	PVC	PI	BP [14]	Silicon oil
d (μm)	130	25	33	/
ε	4.7	3.45	5	2.7
σ (S/m)	8.85×10^{-11}	1.76×10^{-15}	2.8×10^{-11}	5×10^{-12}

Supplementary References

1 Allen, H. G. *Analysis and Design of Structural Sandwich Panels: The Commonwealth and International Library: Structures and Solid Body Mechanics Division* (Elsevier, 2013).

2 Sheng, P. & Wen, W. Electrorheological fluids: mechanisms, dynamics, and microfluidics applications. *Annu. Rev. Fluid Mech.* **44**, 143-174 (2012).

3 Xu, Y., Burdet, E. & Taghavi, M. Electromechanical model for electro-ribbon actuators. *Int. J. Mech. Sci*. **275** (2024).

4 Sun, J. *et al.* Self-powered in-phase sensing and regulating mechanical system enabled by nanogenerator and electrorheological fluid. *Adv. Funct. Mater.* **33**, 2212248 (2023).

5 Fung, Yuan-cheng. *Biomechanics: Mechanical Propertis of Living Tissues* (Springer Science & Business Media, 2013).

6 Reynolds, D., Repperger, D., Phillips, C., & Bandry, G. Modeling the dynamic characteristics of pneumatic muscle. *Ann. Biomed. Eng.* **31**, 310-317 (2003).

7 Wen, W., Huang, X., Yang, S., Lu, K. & Sheng, P. The giant electrorheological effect in suspensions of nanoparticles. *Nat. Mater.* **2**, 727-730 (2003).

8 Do, B. H., Choi, I. & Follmer, S. An all-soft variable impedance actuator enabled by embedded layer jamming. *IEEE/ASME Trans. Mechatron.* **27**, 5529-5540 (2022).

9 Choi, I. *et al.* A soft, controllable, high force density linear brake utilizing layer jamming. *IEEE Rob. Autom. Lett.* **3**, 450-457 (2017).

10 Li, W.-B., Zhang, W.-M., Zou, H.-X., Peng, Z.-K. & Meng, G. Bioinspired variable stiffness dielectric elastomer actuators with large and tunable load capacity. *Soft Rob.* **6**, 631-643 (2019).

11 Levine, D. J., Turner, K. T. & Pikul, J. H. Materials with electroprogrammable stiffness. *Adv. Mater*. **33**, 2007952 (2021).

12 Shintake, J., Schubert, B., Rosset, S., Shea, H. & Floreano, D. In *2015 IEEE/RSJ International Conference on Intelligent Robots and Systems (IROS).* 1097-1102 (IEEE, 2015).

13 Li, Y., Chen, Y., Yang, Y., & Wei, Y. Passive particle jamming and its stiffening of soft robotic grippers. *IEEE Trans. Robot.* **33**, 446-455 (2017).

14 Sîrbu, I. D. *et al.* Electrostatic actuators with constant force at low power loss using matched dielectrics. *Nat. Electron.* **6**, 888-899 (2023).

15 Taghavi, M., Helps, T. & Rossiter, J. Electro-ribbon actuators and electro-origami robots. *Sci. Rob.* **3**, eaau9795 (2018).

16 Suo, Z. Theory of dielectric elastomers. *Acta Mech. Solida Sin.* **23**, 549-578 (2010).

17 Li, Y., Chen, Y., Ren, T., & Hu, Y. Passive and active particle damping in soft robotic actuators. *in 2018 IEEE International Conference on Robotics and Automation (ICRA).* 1547-1552 (IEEE, 2018).

18 Wei, Y., Chen, Y., Ren, T., Chen, Q., Yan, C., Yang, Y., & Li, Y. A novel, variable stiffness robotic gripper based on integrated soft actuating and particle jamming. *Soft Rob.* **3**, 134-143 (2016).

19 Zhao, Y., & Meng, G. A bio-inspired semi-active vibration isolator with variable-stiffness dielectric elastomer: Design and modeling. *J. Sound. Vib.* **485**, 115592 (2020).

20 Liu, Y., Matsuhisa, H., & Utsuno, H. Semi-active vibration isolation system with variable stiffness and damping control. *J. Sound. Vib.* **313**, 16-28 (2008).

21 Sun, S., Yang, J., Li, W., Deng, H., Du, H., & Alici, G. Development of a novel variable stiffness and damping magnetorheological fluid damper. *Smart Mater. Struct.* **24**, 085021 (2015).
