# Supplementary material for: Monolithic electrostatic actuators with independent stiffness modulation
Source: Nat Commun. 2025 Jan 30;16:1174. doi: 10.1038/s41467-025-56455-z (PMC11782490; doi:10.1038/s41467-025-56455-z)
Supplement: Supplementary file 2 — Description of Additional Supplementary Files [file 41467_2025_56455_MOESM2_ESM.docx]

**Description of Additional Supplementary Files**

Supplementary Movie 1. ESRA-supported beam oscillation.

1. The arm supported by a standard ESRA is impacted by an 8.8 g object free-falling onto the arm’s tip. The applied stiffening voltage are 0 kV and 7 kV, respectively.
2. The arm supported by a standard ESRA undergoes an initial displacement, pulled down by a rope and subsequently released. The applied stiffening voltages are 0 kV and 7 kV, respectively.

Supplementary Movie 2. ESRA-actuated beam under load impact.

1. The arm, actuated by a standard ESRA, is hit by a released 24.7 g object. The actuation voltage is 7 kV, with stiffening voltages of 0 kV and 7 kV, respectively.
2. The arm, actuated by a standard ESRA, is hit by a released 11.3 g object. The actuation voltage is 7 kV, with a stiffening voltages of 0 kV and 7 kV, respectively.

Supplementary Movie 3. ESRA-driven musculoskeletal arm model.

1. Standard actuation mode of ESRA. Musculoskeletal model actuated by ESRA with an actuation voltage of 7 kV.
2. Stiffness-enhanced actuation mode of ESRA. An 11.3 g load is positioned at 108 mm and released once the arm reaches this height. The actuation voltage is set to 7 kV with stiffening voltage of 0 kV and 7 kV, respectively.

Supplementary Movie 4. Antagonistic musculoskeletal arm model with ERA and ESRA.

The ventral ERA in the upper arm functions as an actuator for arm elevation, and the dorsal ESRA serves as a resistance element. The actuation voltage is 6kV with stiffening voltage of 0 kV and 7 kV, respectively.

Supplementary Movie 5. Dual ESRAs for adjustable stiffness and damping.

Two ESRAs in series with a load. A 5 kV sine wave excites the lower actuator. For high stiffness, a 5 kV stiffening voltage is applied to the upper actuator and 0 kV to the lower. For high damping, a 0 kV stiffening voltage is applied to the upper and 5 kV to the lower actuator.
